# Supplementary material for: Origin of the quasi-quantized Hall effect in ZrTe5
Source: Nat Commun. 2021 May 27;12:3197. doi: 10.1038/s41467-021-23435-y (PMC8159947; doi:10.1038/s41467-021-23435-y)
Supplement: Supplementary file 1 — Supplementary Information [file 41467_2021_23435_MOESM1_ESM.pdf]

Supplementary Information for the manuscript

Origin of the quasi-quantized Hall effect in $ZrTe_5$

Authors

S. Galeski[1]*, T. Ehmcke[2], R. Wawrzyńczak[1], P. M. Lozano[3], K. Cho[4], A. Sharma[4], S. Das[4], F. Küster[4], P. Sessi[4], M. Brando[1], R. Küchler[1], A. Markou[1], M. König[1], P. Swekis[1]. C. Felser[1], Y. Sassa[5], Q. Li[3], G. Gu[3], M. v. Zimmermann[7], O. Ivashko[7], D.I. Gorbunov[8], S. Zherlitsyn[8], T. Förster[8], S. S. P. Parkin[4], J. Wosnitza[8, 9], T. Meng[2], J Gooth[1,9#]

Affiliations

[1]*Max Planck Institute for Chemical Physics of Solids, Nöthnitzer Straße 40, 01187 Dresden, Germany.*

[2]*Institute of Theoretical Physics, Technische Universität Dresden, 01062 Dresden, Germany.*

[3]*Condensed Matter Physics and Materials Science Department, Brookhaven National Laboratory, Upton, NY, USA.*

[4]*Max Planck Institute of Microstructure Physics, Weinberg 2, 06120 Halle (Saale), Germany.*

[5]*Department of Physics, Chalmers University of Technology, SE-412 96 Gothenburg, Sweden.*

[6]*Institut für Festkörper- und Materialphysik, Technische Universität Dresden, 01062 Dresden, Germany.*

[7]*Deutsches Elektronen-Synchrotron DESY, Notkestraße 85, D-22607 Hamburg, Germany*

[8]*Hochfeld-Magnetlabor Dresden (HLD-EMFL) and Würzburg-Dresden Cluster of Excellence ct.qmat, Helmholtz-Zentrum Dresden-Rossendorf, 01328 Dresden, Germany.*

[9]*Institut für Festkörper- und Materialphysik, Technische Universität Dresden, 01062 Dresden, Germany.*

Table of Content

Supplementary Note 1: Charge-carrier density and mobility from Hall measurements

Supplementary Note 2: Mapping of the Fermi surface by analyzing Shubnikov-de Haas oscillations

Supplementary Note 3: Calculation of the Hall conductivity tensor element

Supplementary Note 4: Non-linear electrical transport in multilayer quantum Hall systems

Supplementary Note 5: Derivative relations between electrical and thermoelectrical quantum transport coefficients in $ZrTe_5$

Supplementary Note 6: Discussion of the sensitivity of Electrical resistivity, thermoelectric coefficients and magnetization for probing the hypothetical Charge-Density-Wave in $ZrTe_5$

Supplementary Note 7: Discussion of the sensitivity of Scanning Tunneling Spectroscopy for probing the hypothetical Charge-Density-Wave in $ZrTe_5$

Supplementary Note 8: Discussion of the sensitivity of ultrasound propagation measurements for probing the hypothetical Charge-Density-Wave in $ZrTe_5$

Supplementary Note 9: Discussion of the sensitivity of X-ray diffraction for probing the hypothetical Charge-Density-Wave in $ZrTe_5$

Supplementary Note 10: Discussion of the sensitivity of Raman spectroscopy for probing the hypothetical Charge-Density-Wave in $ZrTe_5$

Supplementary Fig. 1 – 17

Supplementary Table 1

Supplementary References

Supplementary Note 1: Charge-carrier density and mobility from Hall measurements

From linear fits of the low-field Hall measurements (Supplementary Fig. 2a and b), we obtain the temperature-dependent dominant charge-carrier concentration $n = (\mathrm{d}\rho_{xy}/\mathrm{d}|B|\cdot e)^{-1}$ and the average mobility $\mu = (\rho_{xx,0}\cdot e\cdot n)^{-1}$ (Supplementary Fig. 2c) of $ZrTe_5$, using a single-band model.

Supplementary Note 2: Mapping of the Fermi surface by analyzing Shubnikov-de Haas oscillations

We mapped the Fermi surface of our $ZrTe_5$ samples by analyzing Shubnikov-de Haas oscillations in the temperature (T)-dependent longitudinal magneto-electrical resistivity $\rho_{xx}(\boldsymbol{B})$.[1] In these measurements, the electrical current is applied along the *a*-axis with the magnetic field set along the *a*, *b,* and *c* axis of the crystals. The results of our analysis are summarized in Supplementary Table 1. For all directions, we observe single frequencies $B_{F,i}$, as shown in (Figs. 1 – 3 and Supplementary Figs. 3 - 14) with *i* being the direction of applied magnetic field. The Landau level index v is related to the Fermi surface as $2\pi(v + g) = B_{F,j}/\boldsymbol{B}$, where the phase shift g is approximately zero for all samples. From the slope of linear fits in Landau-index fan diagrams, we extract $B_{F,j}$ and use the Onsager relation $B_{F,j} = (\hbar/2\pi e)S_{F,j}$ to extract the Fermi surface cross-section $S_{F,j}$. Here, $\hbar$ is the reduced Planck constant and e is the electron charge. Under the assumption of an ellipsoidal Fermi surface, the Fermi wave vectors

are then given by $k_{F,a} = \sqrt{S_{F,b}S_{F,c}}/\sqrt{\pi S_{F,a}}$, $k_{F,b} = \sqrt{S_{F,a}S_{F,c}}/b$ and $k_{F,c} = \sqrt{S_{F,a}S_{F,b}}/\sqrt{\pi S_{F,c}}$. The $k_{F,j}$ relate then directly to the Fermi wave length $\lambda_{F,j} = 2\pi/k_{F,j}$.

The resistance amplitude of the maxima in the Shubnikov-de Haas oscillations in the oscillatory part of the longitudinal electrical resistivity $\Delta\rho_{xx}(\boldsymbol{B})$ is proportional to $\chi(\boldsymbol{B})/\sinh[\chi(\boldsymbol{B})]\cdot\exp(\omega_c\tau/\boldsymbol{B})$ with the cyclotron frequency $\omega_{c,j} = \frac{e\,|\boldsymbol{B}|}{m_{c,j}}$ and $\chi(\boldsymbol{B}) = \frac{2\pi^2 k_B T m_c}{\hbar e|\boldsymbol{B}|}$, where $m_{c,j}$ is the cyclotron mass. Hence, when plotting $\log(\Delta\rho_{xx}(\boldsymbol{B})\cdot\chi(\boldsymbol{B})/\sinh[\chi(\boldsymbol{B})])$ against $1/\boldsymbol{B}$, the carrier lifetime τ can be extracted from the slope of the logarithmic (Supplementary Fig. 8c-e). $\Delta\rho_{xx}(\boldsymbol{B})$ is obtained from subtracting the smooth background from the measurement data using a 2nd order polynomial. The corresponding effective mass can be extracted from fitting the T-dependence to $\chi(\boldsymbol{B})/\sinh[\chi(\boldsymbol{B})]$ (Supplementary Fig. 8f-h). Assuming that the massive Dirac band exhibits a linear dispersion at low energies, we finally can obtain the effective masses m^* from the cyclotron masses in the x, y and z direction: $m_{c,a} = \sqrt{m_b^* m_c^*}$, $m_{c,c} = \sqrt{m_a^* m_b^*}$ and $m_{c,b} = \sqrt{m_a^* m_c^*}$, respectively. The Fermi velocities $v_{F,j}$ can be further obtained with $v_{F,j}\, m^*_j = \hbar\, k_{F,j}$. Eventually, the average Fermi energy can be estimated using $E_F = (v_{F,a}^2\, \hbar^2 k_{F,a}^2 + v_{F,b}^2\, \hbar^2 k_{F,b}^2 + v_{F,c}^2\, \hbar^2 k_{F,c}^2)^{0.5}$. For sample A we obtain $E_F = (12 \pm 3)$ meV, in agreement with the distance of E_F and the conduction band edge CB_1 observed in the scanning tunneling spectra in Fig. 2e. The deviation of E_F is obtained from the error of the fits in $k_{F,i}$ and $v_{F,i}$.

Supplementary Note 3: Calculation of the Hall conductivity tensor element

We calculate the Hall conductivity tensor element σ_{xy} using $\sigma_{xy} = \rho_{xy}/(\rho_{xx}^2+\rho_{xy}^2)$, assuming that $\rho_{xx} = \rho_{yy}$. However, in general $\sigma_{xy} = \rho_{xy}/(\rho_{xx}\,\rho_{yy} + \rho_{xy}^2)$ with a magnetic field in b-direction. Due to the geometry of the $ZrTe_5$ crystals (elongated needles) and its mechanical fragility, performing reliable measurements of ρ_{yy} on our samples is not possible. Instead, we estimate

the error of the σ_{xy} using the ratio of Drude resistivities $\rho_{yy}/\rho_{xx} = (n_{ac}e^2\tau_a/\boldsymbol{m_a^*})/\ (n_{ac}e^2\tau_c/\boldsymbol{m_c^*})$ estimated from the quantum lifetimes and effective masses obtained from Shubnikov-de Haas oscillations on sample A, given in Supplementary Table 1. n_{ac} is the charge-carrier concentration in the x-y-plane. Based on this analysis we find $\rho_{yy}/\rho_{xx} \approx 0.8$, which results in an error of 1 % between the theoretical σ_{xy} and ρ_{xy} at the Hall plateaus, owing to $\rho_{xx}(\boldsymbol{B}) < \rho_{xy}(\boldsymbol{B})$. This error lays well within the estimated error of $k_{F,b}$ of 10 %. Therefore, the scaling of the Hall plateaus in σ_{xy}. Hall plateaus are expected to be observable in the measurement of in ρ_{xy}.

Supplementary Note 4: Non-linear electrical transport in multilayer quantum Hall systems

In Ref.[1], a non-Ohmic transport behavior has been observed in the quantum Hall state, interpreted as evidence for the existence of a magnetic field-induced charge density wave (CDW). Indeed, one expects a non-Ohmic behavior arising from a sliding CDW state when the applied bias voltage or current reaches its depinning threshold. However, non-Ohmic transport is also seen in multilayer quantum Hall systems without a CDW[2] - or more general, without a sliding of the 2DEG lattice. Instead, the non-Ohmic current–voltage characteristics is caused by a crossover from surface transport to bulk transport upon reaching a certain threshold voltage: At low voltages, the current flows through the surface of the sample only, but at high voltages also through its bulk when localization gaps are overcome. Hence, a mon-Ohmic transport characteristics does not necessarily provide evidence for a CDW state in a quantum Hall system.

Supplementary Note 5: Derivative relations between electrical and hermoelectrical quantum transport coefficients in ZrTe5

For a wide range of conditions, high mobility two-dimensional quantum Hall systems have been observed to display the empirical electrical[3–6] and thermoelectrical[7] derivative relations

$$\rho_{xx} = \gamma_r B \, d\rho_{xy}/dB, \qquad (1)$$

and

$$S_{xy} = \gamma_s B \, dS_{xx}/dB, \qquad (2)$$

where B is the magnetic field, ρ_{xx} and ρ_{xy} are the longitudinal and Hall component of the resistivity tensor, respectively, and S_{xy} and S_{xx} are the Nerst and the Seebeck coefficient, respectively. γ_r and γ_s are sample-dependent constants, found to be in the range of 0.01 – 0.05 and approximately equal to each other. They provide a measure of the local electron concentration fluctuations,[8–10] determining the dissipation in the quantum Hall system.

To test these relations for the 3D Hall effect in $ZrTe_5$, we have plotted both the measured and calculated (Eq. 1) ρ_{xx} for Sample A (Supplementary Fig. 15 a) and Sample B (Supplementary Fig. 15 b) as well as the measured and calculated (Eq. 2) S_{xy} for Sample A (Supplementary Fig. 15 c) and Sample B (Supplementary Fig. 15 d). We find that the measured and calculated quantities show maxima and minima at the same magnetic field positions. In particular, the derivative relations are well fulfilled with $\gamma_r = 0.04$ and $\gamma_s = 0.01$ for Sample A and $\gamma_r = 0.03$ and $\gamma_s = 0.01$ for Sample B, which is in the expected range reported for two-dimensional quantum Hall systems. These results indicate that the plateaus observed in ρ_{xy} of $ZrTe_5$, are indeed related to quantum Hall physics.

Supplementary Note 6: Discussion of the sensitivity of Electrical resistivity, thermoelectric coefficients and magnetization for probing the hypothetical Charge-Density-Wave in $ZrTe_5$

Electrical resistivity, thermoelectric coefficients and magnetization on their own might not be enough to conclude the absence of a charge-density-wave (CDW) transition. Nevertheless, we argue that despite the backgrounds in each of these quantities, we clearly resolve Shubnikov-de Haas and de Haas van-Alphen oscillations up to the 4th and 5th Landau level. The ability to see such tiny modifications of the density of states in a 3D system at the Fermi level strongly supports that the resolution of all of these measurements is well in range to detect a full gapping of the Fermi surface as expected for the CDW scenario in the quantum limit of $ZrTe_5$.

Quantitatively, the expected effect of a potential CDW transition in $ZrTe_5$ on the electrical resistivity, thermoelectric coefficients and magnetization in fields at low temperatures can be estimated from the low or zero-field temperature-dependencies of these quantities, because $ZrTe_5$ undergoes a Lifshitz transition at around 90 K. At this Lifshitz transition, the Fermi level crosses the intrinsic band gap of $ZrTe_5$ between hole and electron bands, which is in the order of 20 meV[1] and, hence, of the order of the expected CDW gap[11] in the magnetic field range of interest at low temperatures. Each of the quantities, electrical resistivity, thermoelectric coefficients and magnetization, shows a significant response at the Lifshitz transition as explained in the main text of the manuscript. It is therefore to be expected that the opening of a CDW gap, if it is of the size that was stipulated by papers advocating this scenario,[1,11] should similarly show up in these measurements.

Supplementary Note 7: Discussion of the sensitivity of Scanning Tunneling Spectroscopy for probing the hypothetical Charge-Density-Wave in $ZrTe_5$

Scanning Tunneling Spectroscopy (STS) provides direct information about the electronic density of states in a sample as a function of the energy and is probes the local electronic structure of metals, semiconductors, insulators and CDW gaps on a scale unobtainable with other spectroscopic methods.[12–14] We do not see a reason why STS should be not be able to detect the CDW formation in $ZrTe_5$.

Quantitatively, the expected effect of a potential CDW transition in $ZrTe_5$ on the dI/dV spectra at low temperatures can be estimated from the bulk band gap observed as a drop of the dI/dV magnitude above CB_1 in Fig. 2e, which is in the order of 20 meV[1] and, hence, of the order of the expected CDW gap[11] in the magnetic field range of interest at low temperatures. It is therefore to be expected that the opening of a CDW gap, if it is of the size that was stipulated by papers advocating this scenario,[1,11] should similarly show up in these measurements. Still, no signatures of a CDW were observed in our measurements.

Supplementary Note 8: Discussion of the sensitivity of ultrasound propagation measurements for probing the hypothetical Charge-Density-Wave in $ZrTe_5$

As mentioned in the main text, ultrasound propagation is considered one of the most sensitive probes for detecting phase transitions. One argument for this sensitivity is that ultrasound measurements probe the system's compressibility, and thus a thermodynamic quantity that is very much sensitive to sudden changes in the free energy, such as those caused by a CDW gap opening. This idea has been successful applied in a wide range of materials at the CDW transition.[15–21] We do not see a reason why ultrasound should be not be able to detect the CDW formation in $ZrTe_5$. Still, no signatures of a CDW were observed in our measurements.

For a quantitative estimate of the resolution of our ultrasound measurement setup, we recall that for usual electronic phase transitions the changes of sound velocity are in the order of 10^{-3} - 10^{-2}. The setup used in our experiment can detect changes of sound velocity of the order of 10^{-6} down to 2 K and magnetic fields up to 38 T.[22] Our measurement could thus only miss the CDW formation if the electron-phonon coupling is extremely weak, and if the CDW gap is particularly small, which would in turn directly contradict the existing theoretical picture for the formation of a CDW in $ZrTe_5$ proposed in Supplementary Ref. 11.

To test if we can instead reconcile our ultrasound data with our theoretical scenario, we modelled the ultrasound measurement as follows. We started from the exact same model with the exact same model parameters as were used for the description of transport and magnetization, to which we added a generic electron-phonon-coupling. We then calculated the sound velocity renormalization to second order in the electron-phonon-coupling. The sound velocity renormalization more precisely derives from an electronic particle-hole bubble diagram. In that respect, our calculation is very similar to the one of Ref. [23] for Weyl semimetals. We find that the changes of the sound velocity in $ZrTe_5$ observed in our experiments on samples can well be explained in our model (see Fig. 2f).

Supplementary Note 9: Discussion of the sensitivity of X-ray diffraction for probing the hypothetical Charge-Density-Wave in $ZrTe_5$

X-ray diffraction (XRD) is a very sensitive tool for the study of structural properties of matter. An XRD experiment can, therefore, typically detect the formation of a CDW via the emergence of a satellite Bragg peaks together with an additional broad fluctuation contribution close to the transition point.[14] Those additional satellite peaks are expected to originate from both the charge

modulation of the electrons involved in the formation of the CDW order and the additional modulation of charge density of the core electrons associated with the modulation of the lattice.

The reason we have selected the (010)-reflection as a benchmark for the search of satellite reflections is that in orthorhombic crystals the (010)-reflections are forbidden by selection rules. Here, their presence signifies a slight distortion from the perfect orthorhombic symmetry. The observed (010)-type reflections are over 200-times smaller than the main Bragg reflections in the case of $ZrTe_5$.[24] This fact alone signifies that the intensity of the used beam allows to resolve even the slightest distortion to perfect periodicity. In addition, the faint appearance of the (010)-type reflections ensures that the potentially emerging satellite peaks would not be overshadowed by the main reflection.

For a quantitative estimate of the momentum (k) resolution of our X-ray diffraction measurements, we use the rather conservative assumption that the apparent width of the (010)-reflection is already limited by instrumental resolution. This procedure puts a lower bound on our k-space resolution to be ca. 0.036 nm^{-1}. This is an order of magnitude smaller than the expected k_q of the CDW in $ZrTe_5$.[1]

Of course, there still remains the argument that the amplitude of charge modulation could be so small that the new Bragg reflections would not be observable despite using an X-Ray source even with very high brilliance (P21 beamline at Petra III, DESY Hamburg). Recently, Qin et al.[11] have used the band structure parameters of $ZrTe_5$ to calculate the required strength of the phonon-electron coupling, and the energy gap resulting from the formation of a CDW in this model. They estimated that the CDW gap should be of the order of 10-20 meV. They also argue that the CDW-formation is driven by electron-phonon-coupling, whose coupling constant would have to be around 537.3eV $\cdot$ nm^{-1}. Those values are significantly bigger than in the corresponding ones for $NbSe_2$ for example, where the gap is only around 5meV.[25] This should

translate to even a greater intensity of the new superstructure Bragg peaks related to the CDW order in $ZrTe_5$. However, already the CDW-induced satellite peaks in the X-ray spectra of $NbSe_2$ are easily detectable even in commercially available in-house X-ray diffractometers.[25] In contrast, our experiments employed synchrotron radiation with incident beam energy 100 keV ensuring we would probe not only the surface of the sample but actually its bulk. In addition, the brilliance of the P.21 beamline exceeds $10^{18}\frac{Photons}{s\,mm^2\,mrad^2 0.1\%\mathrm{BW.}}$ - several orders of magnitude more than achievable in in-house X-ray sources. Such a setting should in our view allow to detect even the smallest structural details (as outlined in the previous sections), in particular partial loss of translational invariance. For diffraction on $NbSe_2$ using in-house x-ray sources see for example Supplementary Ref. 25 and for measurements performed using synchrotron radiation for example Ref. 26.

This suggests that, if present, the CDW order should be clearly visible in our X-ray measurements as additional superstructure satellite peaks. In fact, the same instrument we have used in the study of $ZrTe_5$ has been used in the past for the study CDW order in YBCO[27] with a similar gap size to that expected in $ZrTe_5$. Still, no such peaks were detected in our measurements. We therefore believe that our X-ray data is strongly suggestive of a state without a CDW.

Supplementary Note 10: Discussion of the sensitivity of Raman spectroscopy for probing the hypothetical Charge-Density-Wave in $ZrTe_5$

To demonstrate the sensitivity of our setup, we have also measured on the prototypical CDW material $TiSe_2$, in which we do in fact see the expected additional peaks that emerge due to the formation of a CDW (see Supplementary Fig. 17). In contrast, on $ZrTe_5$, our Raman experiments do not show any signatures of a gap formation (which would be heralded by

additional peaks appearing). Our Raman data is therefore strongly suggesting the absence of a CDW in $ZrTe_5$.

Supplementary Figures

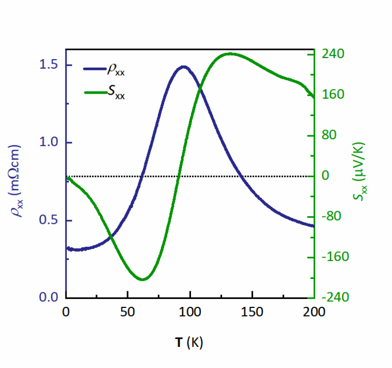

Supplementary Fig. 1. Longitudinal resistivity ρ_{xx} (left axis, dark blue) and Seebeck coefficient (right axis, green) of $ZrTe_5$ sample B as a function of temperature T at zero magnetic field.

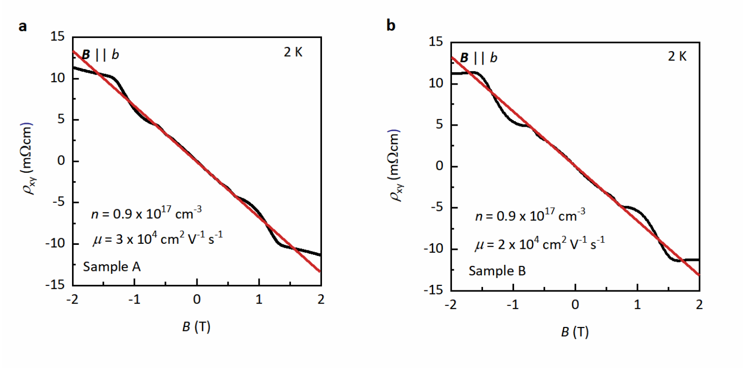

Supplementary Fig. 2. Charge-carrier concentration *n* and Hall mobility μ of sample A and B at 2 K. a, Linear fits (red line) of the Hall resistivity data (black curve) at low magnetic fields (fit range ±0.5 T) of sample A and **b**, of sample B. The charge-carrier concentration $n = (\mathrm{d}\rho_{xy}/\mathrm{d}|\boldsymbol{B}|\cdot e)^{-1}$ is extracted from the slope of the linear fits $\mathrm{d}\rho_{xy}/\mathrm{d}|\boldsymbol{B}|$. Here, e is the electron charge. Subsequently, the Hall mobility $\mu = (\rho_{xx,0}\cdot e\cdot n)^{-1}$ is calculated, using the longitudinal electrical resistivity values at zero field $\rho_{xx,0}$ from Fig. 1a and Supplementary Fig. 1.

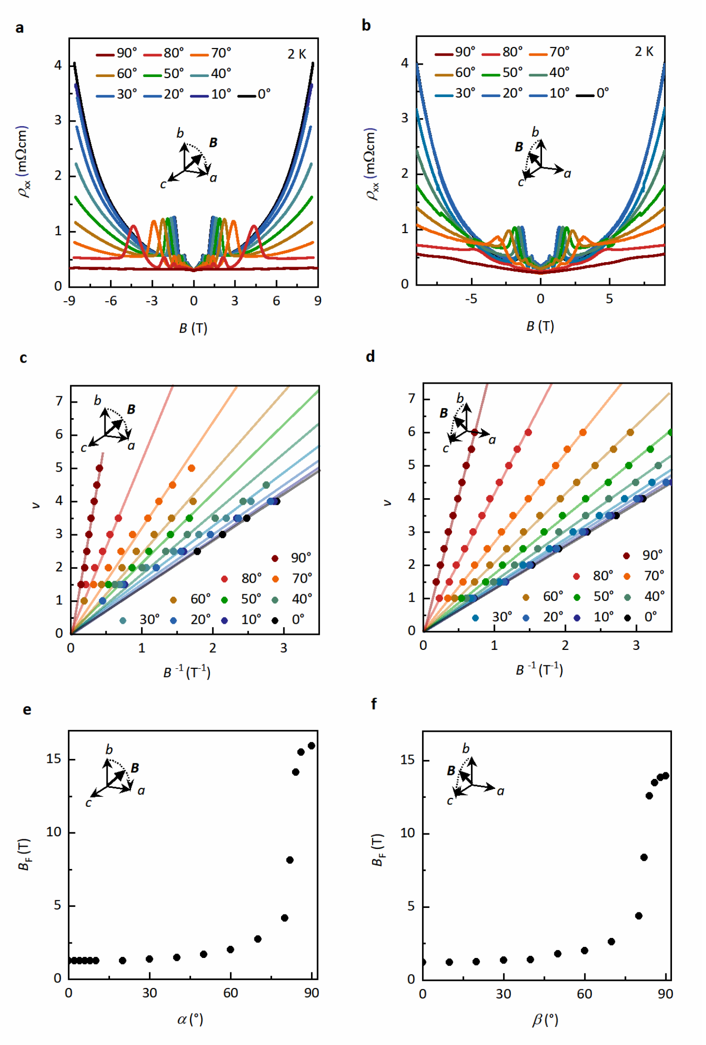

Supplementary Fig. 3. Rotation angle-dependence of the Shubnikov-de Haas oscillations of Sample B. a, The longitudinal electrical resistivity ρ_{xx} versus magnetic field ***B*** for various rotation angles of ***B*** in the *b*-*a* plane and **b**, ρ_{xx} in the *b*-*c* plane at 2 K. **c,** Corresponding Landau-level fan diagram for various rotation angles of ***B*** in the *b*-*a* plane and **d**, in the *b*-*c* plane. The dots in the fan diagrams are the position of the minima (integer Landau index *ν*) and maxima (half-integer *ν*) of ρ_{xx} in versus *B* plotted in Supplementary Fig. 3a and b. The lines are linear fits, which slope is the Shubnikov-de Haas frequency B_F. **e,** B_F as a function of the angle between ***B*** and the *b*-axis, rotated within the *a*-*b* plane and **f**, within the *b*-*c* plane.

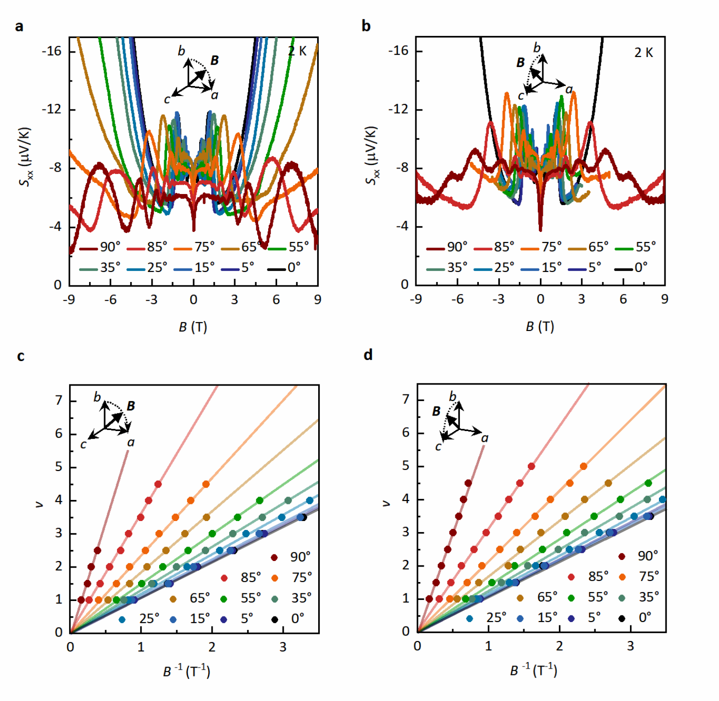

Supplementary Fig. 4. Rotation angle-dependence of the Shubnikov-de Haas oscillations of Sample A. a, The Seebeck coefficient S_{xx} versus magnetic field $\boldsymbol{B}$ for various rotation angles of $\boldsymbol{B}$ in the *b*-*a* plane and **b**, S_{xx} in the *b*-*c* plane at 2 K. **c,** Corresponding Landau-level fan diagram for various rotation angles of $\boldsymbol{B}$ in the *b*-*a* plane and **d**, in the *b*-*c* plane. The points in the fan diagrams are the position of the minima (integer Landau index *ν*) and maxima (half-integer *ν*) of S_{xx} in versus *B* plotted in Supplementary Fig. 3a and b.

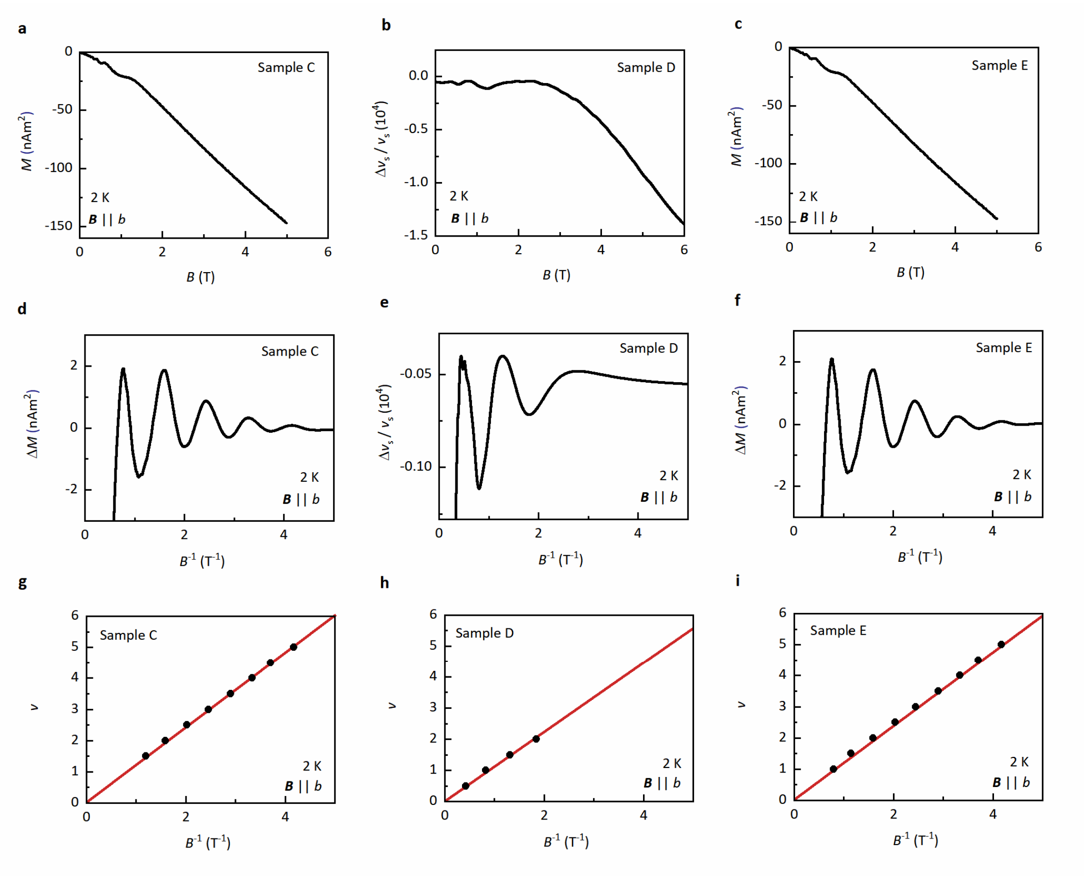

Supplementary Fig. 5. Analysis of the Shubnikov-de Haas oscillations in the thermodynamic properties of Sample C, E and G for the magnetic field *B* applied along the *b*-axis of the crystals at 2 K. a, Magnetization *M* of Sample C as a function of *B*. **b**, Sound velocity variation $\Delta v_S/v_S$ of the transverse mode (propagation along the *a*-axis, polarization vector in the *a*-*c* plane) of Sample E as a function of *B*. **a,** Magnetization *M* of Sample G as a function of *B*. **d**, Oscillatory part of the magnetization ΔM as a function of inverse magnetic field B^{-1}, obtained by subtracting the linear background from the data shown in Supplementary Fig. 5 a. **e**, $\Delta v_S/v_S$ as a function of B^{-1}. **f**, ΔM as a function of B^{-1}, obtained by subtracting the linear background from the data shown in Supplementary Fig. 5 c. **g,** Corresponding Landau-level fan diagram of Sample C; **h**, Sample E; and **i**, Sample G. The points in the fan diagrams are the position of the minima (integer Landau index *ν*) and maxima (half-integer *ν*) the

corresponding data plotted in Supplementary Fig. 5 d-f. The red lines are linear fits, which slope is the Shubnikov-de Haas frequency given in Supplementary Table 1.

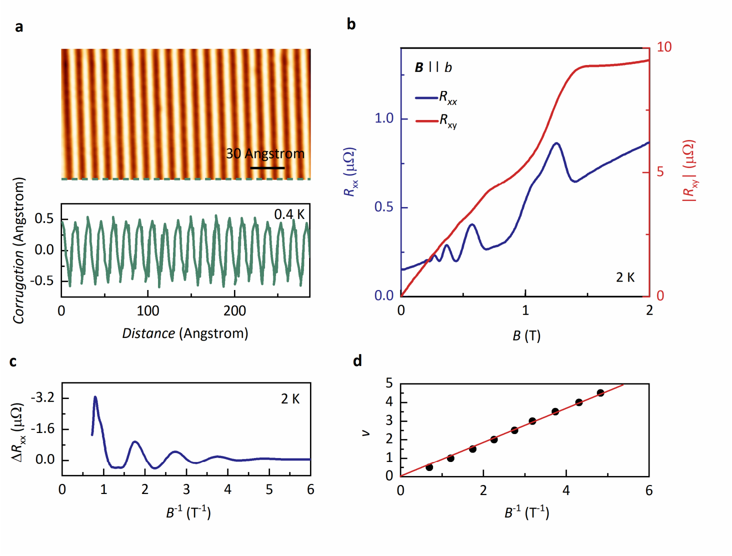

Supplementary Fig. 6. Topography, magnetotransport and analysis of the Shubnikov-de Haas oscillations of Sample D. a, Topography of $ZrTe_5$ from scanning tunneling microscopy at 0.4 K in zero magnetic field B. **b,** Longitudinal electrical resistance R_{xx} (left axis) and Hall resistance R_{xy} (right axis) as a function of B at 2 K with B applied along the b-axis of the crystal. **c**, The oscillatory part of the longitudinal resistance ΔR_{xx} versus inverse magnetic field B^{-1}, obtained by subtracting a second order pominomial background from the R_{xx} data shown in Supplementary Fig. 6 b. **d,** Corresponding Landau-level fan diagram. The points in the fan diagram are the position of the minima (integer Landau index ν) and maxima (half-integer ν). The red line is a linear fit, which slope is the Shubnikov-de Haas frequency given in Supplementary Table 1.

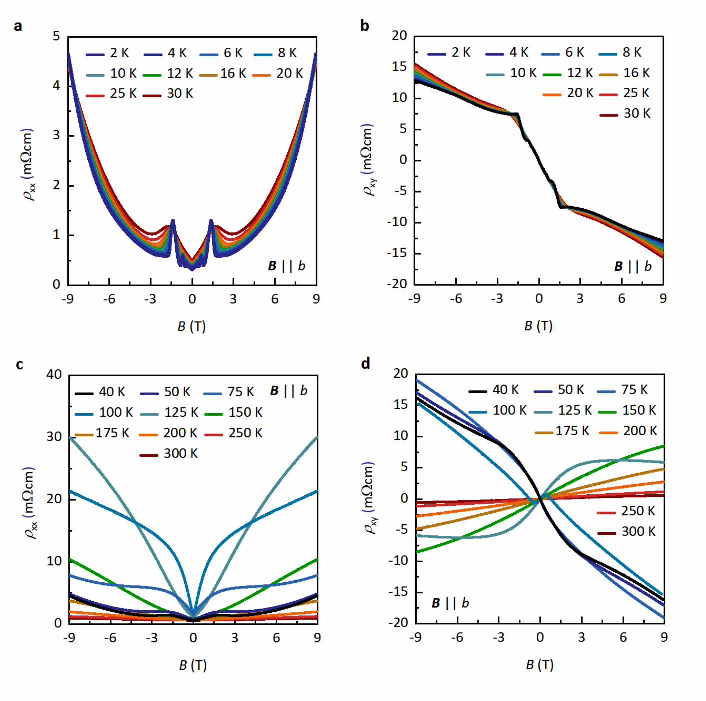

Supplementary Fig. 7. Temperature (*T*)-dependence of the longitudinal electrical resistivity ρ_{xx} and the Hall resistivity ρ_{xy} of Sample B. a, ρ_{xx} and **b**, ρ_{xy} as a function of magnetic field *B* for various *T* (30 K ≥) with B applied along the b-axis of the crystal. **c,** ρ_{xx} and **d**, ρ_{xy} as a function of *B* for various 200 K ≥ *T* > 30 K with *B* applied along the *b*-axis of the crystal.

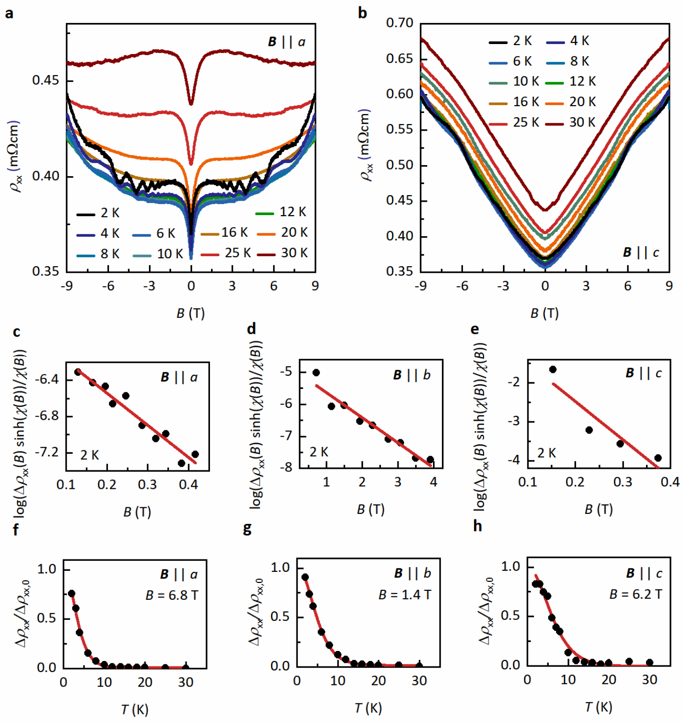

Supplementary Fig. 8. Determination of the mobility and cyclotron mass of Sample B. a, Longitudinal electrical resistivity ρ_{xx} as a function of magnetic field B for various temperatures $T \geq 30$ K with B applied along the a-axis of the crystal and **b**, along the c-axis of the crystal. **c**, Dingle plots of $\Delta\rho_{xx}|B|\sinh[\chi(T)]$ versus B^{-1} with $\chi(B) = \frac{2\pi^2 k_B T m_c}{\hbar e \boldsymbol{B}}$ at 2 K with B applied along the a-axis of the crystal, **d**, the b-axis of the crystal and **e**, along the a-axis of the crystal. The lines are linear fits to the measurement data to obtain the Dingle temperature, as explained in the Supplementary Note 2. **f**, The cyclotron masses m_c for B applied along a, **g**, along b and **h**, along the c-axis of the crystals obtained from fits (red lines) to $\Delta\rho_{xx}/\Delta\rho_{xx,0}$, where $\Delta\rho_{xx,0}$ is the extrapolated oscillatory part of the longitudinal electrical resistivity $\Delta\rho_{xx}$ at zero Kelvin. $\Delta\rho_{xx}$ is obtained by subtracting a second order polynomial background from the measurement data. $\Delta\rho_{xx}$ for each principal direction is taken at the magnetic field of the lowest accessible Landau level.

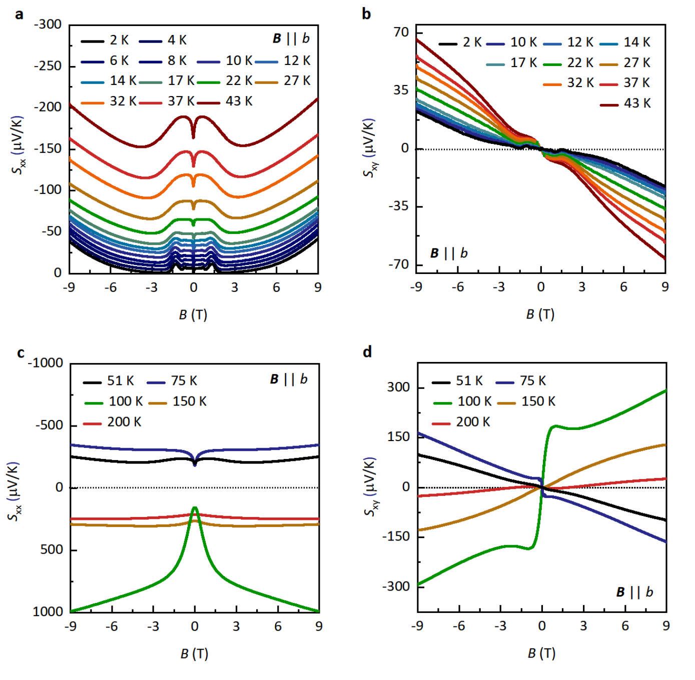

Supplementary Fig. 9. Temperature (*T*)-dependence of the Seebeck coefficient S_{xx} and the Nernst coefficient S_{xy} of Sample A. a, S_{xx} and **b**, S_{xy} as a function of magnetic field *B* for various *T* (42 K ≥) with *B* applied along the *b*-axis of the crystal. **c,** S_{xx} and **d**, S_{xy} as a function of *B* for various 200 K ≥ *T* > 30 K with *B* applied along the *b*-axis of the crystal.

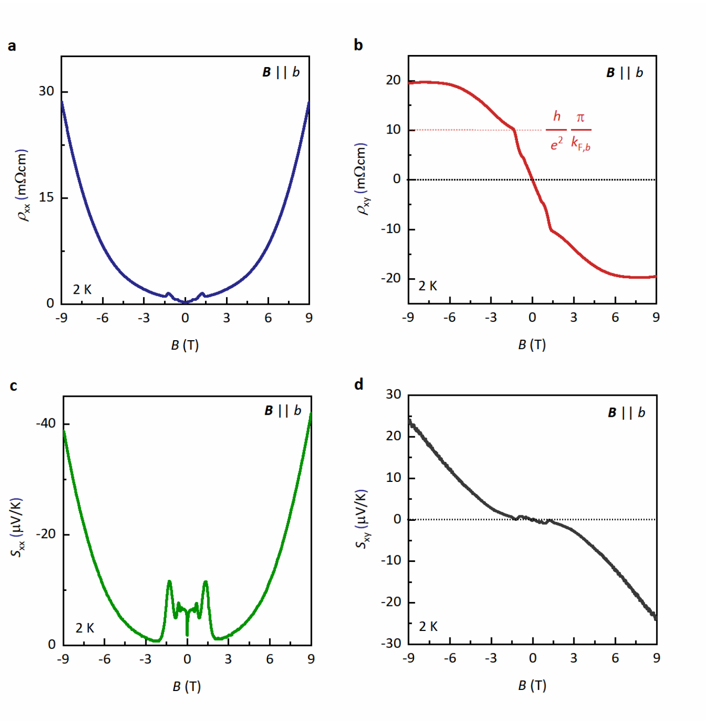

Supplementary Fig. 10. Magneto-transport data of Sample A across the full magnetic field (*B*)-range investigated at 2 K with *B* applied along the *b*-axis of the crystal. a, Longitudinal electrical resistivity ρ_{xx}, **b**, Hall resistivity ρ_{xy}, **c**, Seebeck coefficient S_{xx} and **d**, Nernst coefficient S_{xy} as a function of B. The plateau observed in ρ_{xy} scales with $(h/e^2)\ \pi/k_{F,b}$, with the Planck constant h, the electron charge e, and the Fermi wave vector along the b-axis of the crystal $k_{F,b}$.

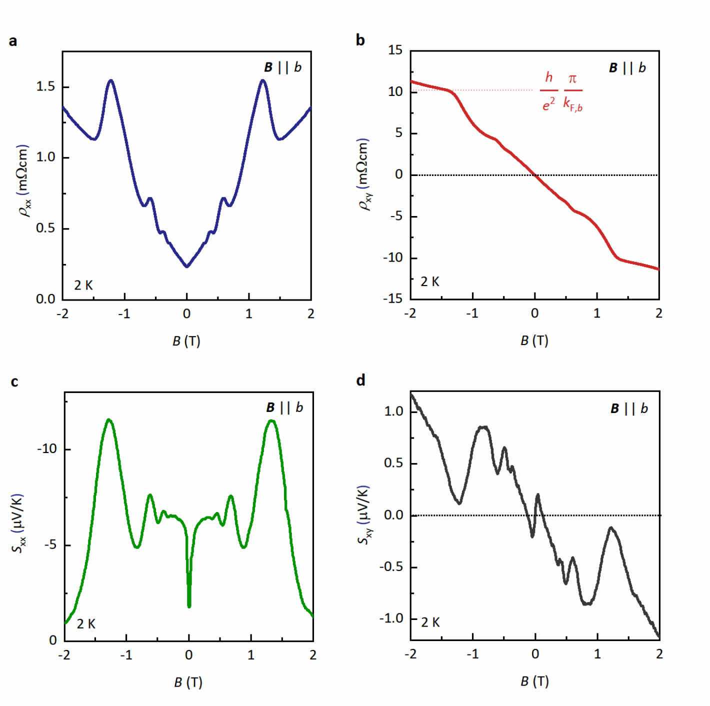

Supplementary Fig. 11. Magneto-transport data of Sample A from -2 T to 2 T at 2 K with the magnetic field *B* applied along the *b*-axis of the crystal. a, Longitudinal electrical resistivity ρ_{xx}, **b**, Hall resistivity ρ_{xy}, **c**, Seebeck coefficient S_{xx} and **d**, Nernst coefficient S_{xy} as a function of B. The plateau observed in ρ_{xy} scales with $(h/e^2)\ \pi/k_{F,b}$, with the Planck constant h, the electron charge e, and the Fermi wave vector along the b-axis of the crystal $k_{F,b}$.

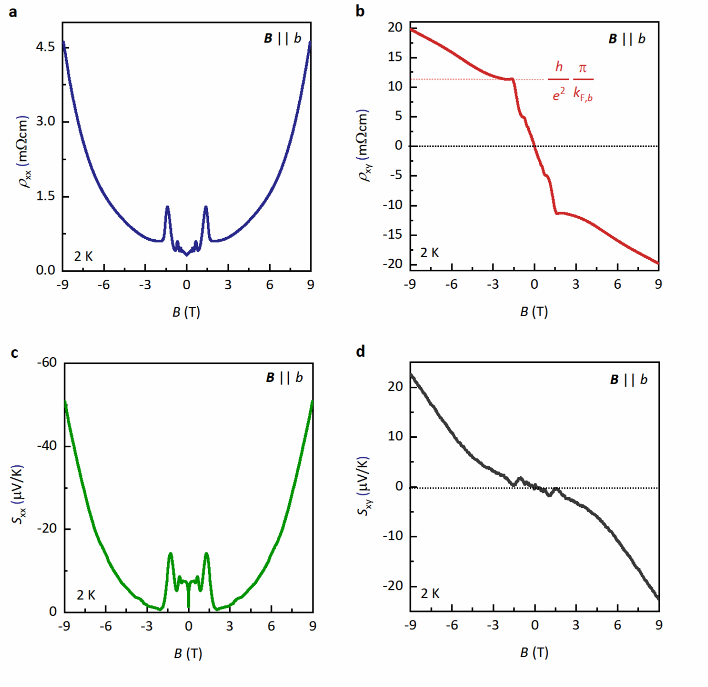

Supplementary Fig. 12. Magneto-transport data of Sample B across the full magnetic field (*B*)-range investigated at 2 K with *B* applied along the *b*-axis of the crystal. a, Longitudinal electrical resistivity ρ_{xx}, **b**, Hall resistivity ρ_{xy}, **c**, Seebeck coefficient S_{xx} and **d**, Nernst coefficient S_{xy} as a function of B. The plateau observed in ρ_{xy} scales with $(h/e^2)\ \pi/k_{F,b}$, with the Planck constant h, the electron charge e, and the Fermi wave vector along the *b*-axis of the crystal $k_{F,b}$.

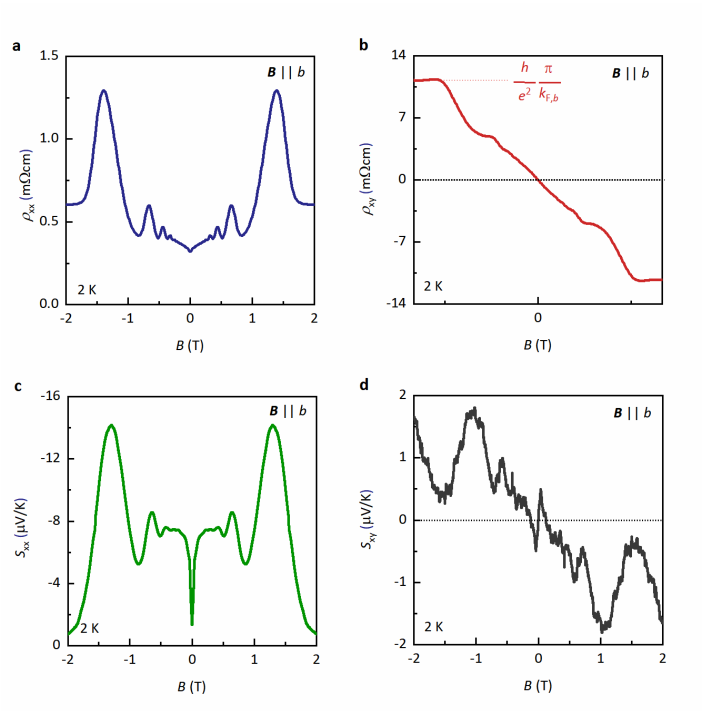

Supplementary Fig. 13. Magneto-transport data of Sample B from -2 T to 2 T at 2 K with the magnetic field *B* applied along the *b*-axis of the crystal. a, Longitudinal electrical resistivity ρ_{xx}, **b**, Hall resistivity ρ_{xy}, **c**, Seebeck coefficient S_{xx} and **d**, Nernst coefficient S_{xy} as a function of B. The plateau observed in ρ_{xy} scales with $(h/e^2)\ \pi/k_{F,b}$, with the Planck constant h, the electron charge e, and the Fermi wave vector along the b-axis of the crystal $k_{F,b}$.

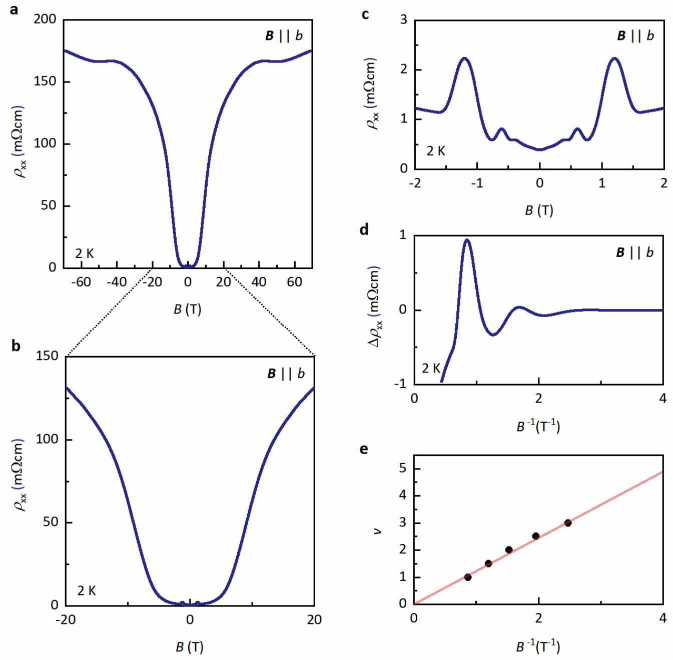

Supplementary Fig. 14. High-field magneto-transport on Sample H. a, Longitudinal electrical resistivity ρ_{xx} at 2 K as a function of magnetic field B applied along the b-axis of the crystal up to ±70 T; **b**, up to ±20 T; and **c**, up to ±2 T. **d**, Oscillatory part of the llongitudinal electrical resistivity $\Delta\rho_{xx}$ as a function of B^{-1}. **e**, Corresponding Landau-level fan diagram. The points in the fan diagram are the position of the minima (integer Landau index v) and maxima (half-integer v) of $\Delta\rho_{xx}$ versus B plotted in Supplementary Fig. 13d.

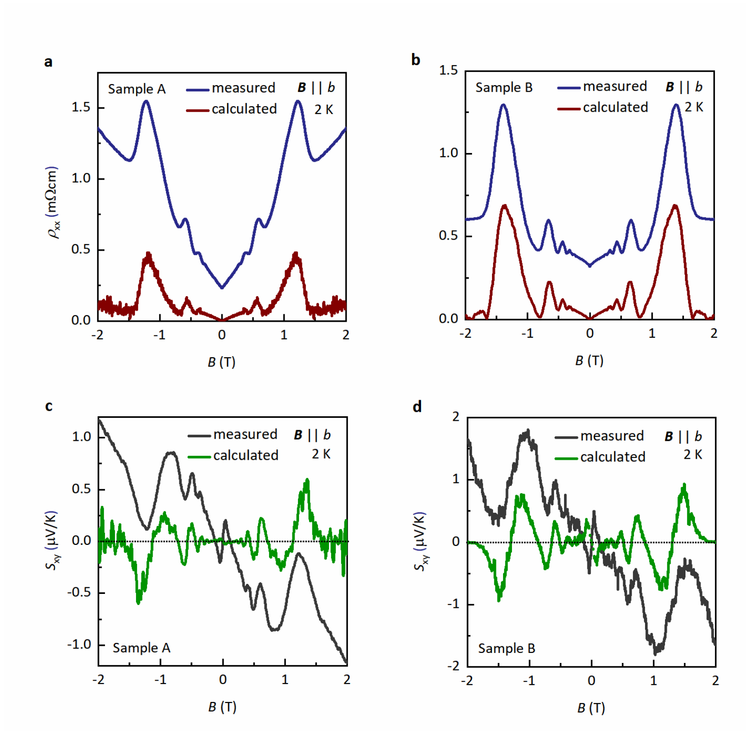

Supplementary Fig. 15. Derivative relations between electrical and thermoelectrical quantum transport coefficients of Sample A and B. a, Measured (blue line) and calculated (red line) longitudinal electrical resistivity ρ_{xx} at 2 K as a function of magnetic field B applied along the b-axis of the crystal of Sample A. The calculated ρ_{xx} is obtained from the empirical relation $\rho_{xx} = \gamma \cdot B \cdot d\rho_{xy}/dB$ from ρ_{xy} plotted in Supplementary Fig. 9 b. **b**, Measured (blue line) and calculated (red line) ρ_{xx} at 2 K as a function of B applied along the b-axis of the crystal of Sample B. The calculated ρ_{xx} is obtained from the empirical relation $\rho_{xx} = \gamma_r \cdot B \cdot d\rho_{xy}/dB$ from ρ_{xy} plotted in Supplementary Fig. 11 b. **c**, Measured (dark grey line) and calculated (green line) Nernst coefficient S_{xy} at 2 K as a function of B applied along the b-axis of the crystal of Sample A. The calculated S_{xy} is obtained from the empirical relation $S_{xy} = \gamma_S \cdot B \cdot dS_{xx}/dB$ from S_{xx} plotted in Supplementary Fig. 9 c. **d**, Measured (dark grey line) and calculated (green line) Nernst coefficient S_{xy} at 2 K as a function of B applied along the b-axis of the crystal of Sample A. The

calculated S_{xy} is obtained from the empirical relation $S_{xy} = \gamma \cdot B \cdot \mathrm{d}S_{xx}/\mathrm{d}B$ from S_{xx} plotted in Supplementary Fig. 11 c. γ_r and γ_s are scaling factors of the order of 0.01.

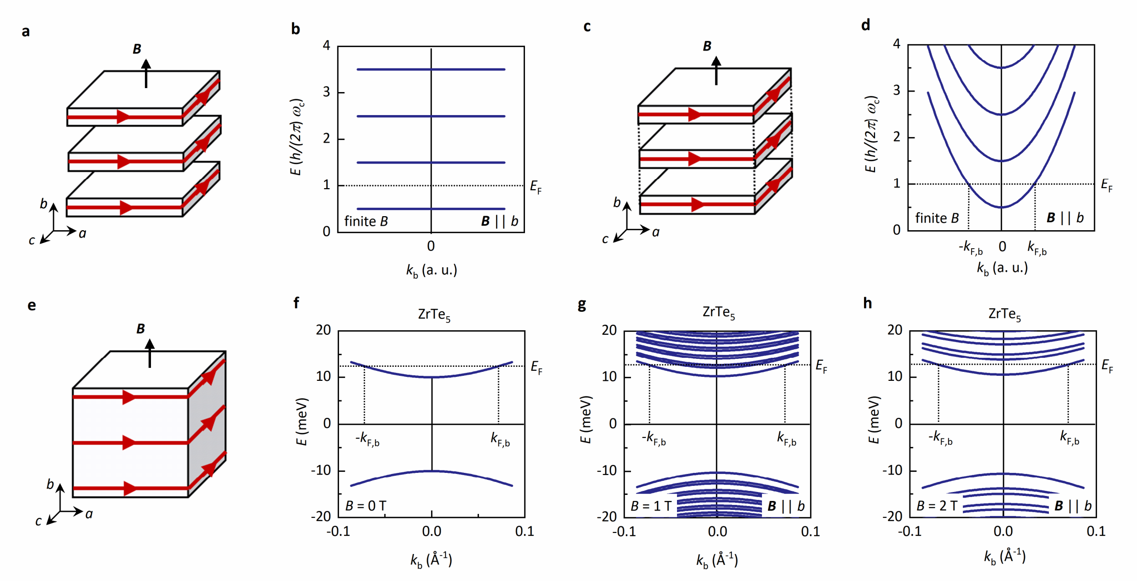

Supplementary Fig. 16. 3D electrons in magnetic fields. a, Sketch of a stack of non-interacting two-dimensional electron systems (2DESs) in the quantum Hall state. *a*, *b* and *c* denote the spatial directions, *B* the magnetic field. **b** Energy (*E*)-levels as a function of momentum vector k_b in *b*-direction of a stack of non-interacting 2DESs in a finite magnetic field. E_F denotes the Fermi level **c**, Sketch of a stack of interacting 2DESs in the quantum Hall state. **d,** Formation of three-dimensional (3D) Landau bands for non-vanishing electron hopping between the layers of a 2DESs. $k_{F,b}$ labels the Fermi wave vector in the *b*-direction. **e,** Sketch of a stack of a 3D electron system in the quantum Hall state. **f**, Dispersion of $ZrTe_5$ around E_F at zero field, **g**, 1T and, **h**, 2 T with the magnetic field applied along the *b*-axis.

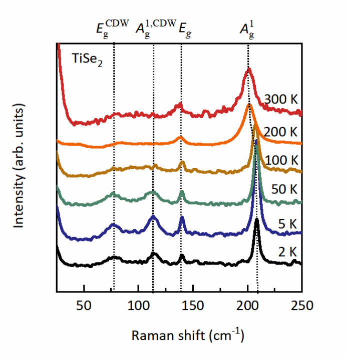

Supplementary Fig. 17. Raman spectra of $TiSe_2$ to bench mark our Raman measurements setup at various temperatures. The Raman modes of the undistorted crystal at high temperatures are located at 136 and 205 cm^{-1}, labelled with E_g, and $A^1{}_g$, respectively. At 205 K exhibits a charge-density wave transition, leading to additional Raman modes at 76 and 115 cm^{-1} labelled with $E_g{}^{CDW}$, and $A^{1,CDW}{}_g$, respectively, in agreement with previous Raman studies on $TiSe_2$.[28,29]

Supplementary Tables

Sample	Magnetic field's direction	SdH frequency B_F (T)	Fermi area S_F (10^{-4} Å^{-2})	Fermi wave vector k_F (10^{-3} Å^{-1})	Fermi wave length λ_F (nm)	Cyclotron mass m_c (m_0)	Effective mass m^* (m_0)	Fermi velocity v_F (10^5 m/s)	Dingle temperature T_D (K)	Lifetime τ (ps)
	B ‖ *a*	16.7 ± 0.5	15.9 ± 0.5	4.8 ± 0.6	130.9 ± 15.1	-	-	-	-	-
A	***B*** ‖ *b*	1.1 ± 0.1	0.9 ± 0.1	77.8 ± 7.9	8.1 ± 0.3	-	-	-	-	-
	B ‖ *c*	12.3 ± 0.3	11.7 ± 0.3	6.8 ± 8.9	96.5 ± 13.8	-	-	-	-	-
	B ‖ *a*	15.2 ± 0.3	15.2 ± 0.3	5.3 ± 1.1	108.1 ± 21.1	0.68 ± 0.02	0.021 ± 0.001	3.1 ± 0.8	0.32 ± 0.03	0.20 ± 0.02
B	***B*** ‖ *b*	1.2 ± 0.2	1.2 ± 0.2	72.9 ± 7.5	8.6 ± 0.9	0.04 ± 0.01	6.078 ± 0.685	0.1 ± 0.03	1.43 ± 0.13	0.91 ± 0.1
	B ‖ *c*	13.9 ± 0.3	13.9 ± 0.3	6.5 ± 1.3	94.4 ± 17.2	0.36 ± 0.08	0.075 ± 0.001	1.0 ± 0.2	1.85 ± 0.54	0.66 ± 0.2
C	***B*** ‖ *b*	1.2 ± 0.1	1.1 ± 0.1	-	-	-	-	-	-	-
D	***B*** ‖ *b*	1.2 ± 0.2	1.2 ± 0.2	-	-	-	-	-	-	-
E	***B*** ‖ *b*	1.0 ± 0.1	0.9 ± 0.1	-	-	-	-	-	-	-
G	***B*** ‖ *b*	1.1 ± 0.2	1.0 ± 0.2	-	-	-	-	-	-	-
H	***B*** ‖ *b*	1.2 ± 0.2	1.1 ± 0.2	-	-	-	-	-	-	-

Supplementary Table 1. Band-structure parameters of $ZrTe_5$ Sample A, B, C, D, E, G and H, obtained from Shubnikov-de Haas oscillations. The variations denote the errors from the fits and from error propagation as explained in the Supplementary Information and the main text.

Supplementary References

1. Tang, F. *et al.* Three-dimensional quantum Hall effect and metal–insulator transition in $ZrTe_5$. *Nature* **569**, 537–541 (2019).

2. Kawamura, M., Endo, A., Katsumoto, S. & Iye, Y. Non-ohmic vertical transport in multilayered quantum hall systems. *Phys. E Low-dimensional Syst. Nanostructures* **6**, 698–701 (2000).

3. Liu, X., Ma, Z. & Shi, J. Derivative relations between electrical and thermoelectric quantum transport coefficients in graphene. *Solid State Commun.* **152**, 469–472 (2012).

4. Chang, A. M. & Tsui, D. C. Experimental observation of a striking similarity between quantum Hall transport coefficients. *Solid State Commun.* **56**, 153–154 (1985).

5. Rötger, T. *et al.* Relation between low-temperature quantum and high-temperature classical magnetotransport in a two-dimensional electron gas. *Phys. Rev. Lett.* **62**, 90 (1989).

6. Stormer, H. L., Baldwin, K. W., Pfeiffer, L. N. & West, K. W. Strikingly linear magnetic field dependence of the magnetoresistivity in high quality two-dimensional electron systems. *Solid State Commun.* **84**, 95–98 (1992).

7. Tieke, B. *et al.* Fundamental relation between electrical and thermoelectric transport coefficients in the quantum Hall regime. *Phys. Rev. Lett.* **78**, 4621 (1997).

8. Simon, S. H. & Halperin, B. I. Explanation for the resistivity law in quantum Hall systems. *Phys. Rev. Lett.* **73**, 3278 (1994).

9. Simon, S. H. & Cooper, N. R. Derivative relation for thermopower in the quantum Hall regime. *Phys. Rev. B* **56**, R7116–R7119 (1997).

10. Endo, A., Hatano, N., Nakamura, H. & Shirasaki, R. Fundamental relation between

longitudinal and transverse conductivities in the quantum Hall system. *J. Phys. Condens. Matter* **21**, 345803 (2009).

11. Qin, F. *et al.* Theory for the charge-density-wave mechanism of 3D quantum Hall effect. *Phys. Rev. Lett.* **125**, 206601 (2020).

12. Güntherodt, H.-J. & Wiesendanger, R. *Scanning tunneling microscopy I: general principles and applications to clean and adsorbate-covered surfaces*. (Springer, 1992).

13. Wiesendanger, R., Güntherodt, H.-J. & Baumeister, W. *Scanning tunneling microscopy II: further applications and related scanning techniques*. (Springer, 1992).

14. Grüner, G. The dynamics of charge-density waves. *Rev. Mod. Phys.* **60**, 1129–1181 (1988).

15. Barmatz, M., Testardi, L. R., Garito, A. F. & Heeger, A. J. Elastic properties of one dimensional compounds. *Solid State Commun.* **15**, 1299–1302 (1974).

16. Saint-Paul, M. *et al.* Elastic anomalies at the charge density wave transition in $TbTe_3$. *Solid State Commun.* **233**, 24–29 (2016).

17. Bourne, L. C. & Zettl, A. Elastic anomalies in the charge density wave conductor K0. 3MoO3. *Solid State Commun.* **60**, 789–792 (1986).

18. Tiedje, T., Haering, R. R., Jericho, M. H., Roger, W. A. & Simpson, A. Temperature dependence of sound velocities in TTF-TCNQ. *Solid State Commun.* **23**, 713–718 (1977).

19. Caillé, A., Lepine, Y., Jericho, M. H. & Simpson, A. M. Thermal expansion, ultrasonic velocity, and attenuation measurements in Ti S_2, Ti Se_2, and $TiS_{0.5}Se_{1.5}$. *Phys. Rev. B* **28**, 5454 (1983).

20. Jericho, M. H. & Simpson, A. M. Effect of electric fields on the propagation of ultrasonic pulses in o-TaS_3. *Phys. Rev. B* **34**, 1116 (1986).

21. Jericho, M. H., Simpson, A. M. & Frindt, R. F. Velocity of ultrasonic waves in 2H-Nb Se_2, 2H-TaS_2, and 1 T-TaS_2. *Phys. Rev. B* **22**, 4907 (1980).

22. Schindler, C. *et al.* Strong anisotropy of the electron-phonon interaction in NbP probed by magnetoacoustic quantum oscillations. *Phys. Rev. B* **102**, 165156 (2020).

23. Zhang, S.-B. & Zhou, J. Quantum oscillations in acoustic phonons in Weyl semimetals. *Phys. Rev. B* **101**, 85202 (2020).

24. Skelton, E. F. *et al.* Giant resistivity and X-ray diffraction anomalies in low-dimensional ZrTe5 and HfTe5. *Solid State Commun.* **42**, 1–3 (1982).

25. Eremenko, V., Sirenko, V., Ibulaev, V., Shvedun, M. & Andre, G. X-ray scattering on charge-density-wave superconductor 2H-$NbSe_2$. in *Journal of Physics: Conference Series (см. в книгах)* **150**, 42029 (Institute of Physics and IOP Publishing Limited, 2009).

26. Chatterjee, U. *et al.* Emergence of coherence in the charge-density wave state of 2 H-$NbSe_2$. *Nat. Commun.* **6**, 1–7 (2015).

27. Chang, J. *et al.* Direct observation of competition between superconductivity and charge density wave order in $YBa_2Cu_3O_6$. *Nat. Phys.* **8**, 871–876 (2012).

28. Snow, C. S., Karpus, J. F., Cooper, S. L., Kidd, T. E. & Chiang, T.-C. Quantum Melting of the Charge-Density-Wave State in 1T− $TiSe_2$. *Phys. Rev. Lett.* **91**, 136402 (2003).

29. Cui, L. *et al.* Raman spectroscopy of optical phonon and charge density wave modes in 1T-$TiSe_2$ exfoliated flakes. *Solid State Commun.* **266**, 21–25 (2017).
